# Socioeconomic inequalities in the quality of care for long-term conditions in England: retrospective, observational study of electronic primary care records from the Clinical Practice Research Datalink

**DOI:** 10.1136/jech-2025-225416

**Published:** 2026-06-12

**Authors:** Tim Doran, Ni Gao, Luigi Siciliani, Simon Mark Walker, Anne Mason, David Glynn, Nils Gutacker

**Affiliations:** 1 Department of Health Sciences, University of York, York, UK; 2 Centre for Health Economics, University of York, York, UK; 3 Department of Economics and Related Studies, University of York, York, UK; 4 School of Engineering, University of Galway, Galway, Ireland; 5 Centre for Health Economics, University of York, Heslington, UK

**Keywords:** GENERAL PRACTICE, PRIMARY HEALTH CARE, Health inequalities

## Abstract

**Background:**

Variation in primary care quality across social groups contributes to health inequalities. We examined achievement rates for quality-of-care indicators among patients attending primary care practices in England, comparing rates for patients living in the most and least deprived areas.

**Methods:**

We analysed electronic health records from 2.2 million patients in 2019/2020, registered with 300 practices contributing to the Clinical Practice Research Datalink (CPRD) Aurum database. Patients were grouped by neighbourhood deprivation using the 2019 Index of Multiple Deprivation. We assessed inequality in 35 quality-of-care indicators from the Quality and Outcomes Framework pay-for-performance scheme for 607 126 patients with a relevant long-term condition, calculating differences in (a) age-sex adjusted achievement rates (overall inequality) and (b) rates further adjusted for common practice fixed effects (within-practice inequality) obtained by logistic regression. Rates were calculated for all patients with relevant conditions (population achievement) and the subset not excluded from the scheme by practices (reported achievement).

**Results:**

There was no consistent socioeconomic pattern in reported achievement of individual indicators overall (across all practices), but within-practice reported achievement was significantly lower for patients in more deprived areas for eight indicators. Population achievement was significantly lower for patients in more deprived areas for 13 indicators, reflecting higher exclusion rates. Population achievement was higher in more deprived areas for one indicator. The largest inequalities were for influenza vaccination (chronic obstructive pulmonary disease and stroke) and haemoglobin A1c (HbA1c) control (diabetes).

**Conclusion:**

Within the same practice, patients from more deprived areas are less likely to receive recommended care for key quality of care indicators.

**WHAT IS ALREADY KNOWN ON THIS TOPIC**

**WHAT THIS STUDY ADDS**

- Using individual-level data linked to area deprivation, we found that within general practices in England, patients living in more deprived areas were less likely to receive some incentivised interventions and were more likely to be excluded from the national pay-for-performance scheme.

**HOW THIS STUDY MIGHT AFFECT RESEARCH, PRACTICE OR POLICY**

- Small but significant socioeconomic inequalities in care persist within general practices in England. Monitoring arrangements and incentive structures may need refinement to support more equitable delivery of care, alongside approaches that recognise social complexity and differences in patients’ ability to engage with incentivised care.

## Introduction

 Health inequalities between social groups are created by variations in socioeconomic conditions with health impacts (including social status, education, occupation and housing)^1^ compounded by variations in access to effective healthcare.^2^ In most low-income and middle-income countries, an ‘inverse care law’ exists, with more socially disadvantaged people tending to receive less high-quality care than more socially advantaged people despite having greater need. In the UK and other high-income countries with universal healthcare, a ‘disproportionate care law’ exists, with socially disadvantaged people tending to receive more care, but still of insufficient quantity and quality to meet their additional needs.^3^ This ‘law’ persists in the UK despite more than two decades of policy efforts to reduce health inequalities, including the introduction of a statutory duty requiring National Health Service (NHS) organisations to address inequalities in both access to care and health outcomes.^4^

Over the same period, primary care in England has been operating under the Quality and Outcomes Framework (QOF), which offers financial incentives to general practices for achieving a range of quality-of-care indicators. This pay-for-performance programme was intended to address the lack of explicit incentives for quality in traditional methods of remuneration (eg, capitation and fee-for-service payments) by linking the financial rewards for physicians to quality of care,^5^ thereby encouraging and supporting practices to improve care for all—or at least the majority—of their patients. By reducing variations in care between practices, pay-for-performance could also reduce inequalities. Conversely, incentives could increase inequalities by providing less healthcare to those with greater health needs,^3^ for example, if providers focus efforts on populations perceived as being easier to treat in preference to populations with more complex health needs requiring greater resources to manage.^6^

In the early years of the QOF, the gaps in recorded performance between practices in more and less deprived areas narrowed^7^ but there was no clear evidence for improved equity of outcomes such as emergency hospital admissions.^8^ After a decade, there was little further reduction in gaps in performance, with practices in less deprived areas on average outperforming practices in more deprived areas.^9^ However, studies of inequalities in care under the QOF have generally been limited by the aggregation of data at the practice level, with the socioeconomic characteristics of the area where the practice is located attributed to all patients attending that practice. This increases the risk of misclassification^10^ and potentially underestimates inequalities. To address this limitation, in this study we link area deprivation data at the level of individual patients, using postcode of residence rather than practice location, to examine: (a) whether achievement of incentivised quality-of-care indicators varies by individual-level socioeconomic deprivation; (b) whether such variation differs when measured across practices compared with within practices; and (c) the extent to which exception reporting contributes to observed socioeconomic differences.

## Methods

We conducted a cross-sectional observational study using routinely collected electronic health records from English general practices.

### Quality and Outcomes Framework

The QOF is a national pay-for-performance scheme introduced across primary care in the UK in April 2004.^11^ It was intended to improve remuneration for general practices while improving population health by incentivising general practitioners (GPs) to deliver care according to agreed best practice standards. GP practices are assessed against a range of annually revised indicators covering secondary management of common long-term conditions such as heart disease, diabetes and asthma.^5^ Practices score ‘points’ for each QOF indicator according to the proportion of their patients for which the indicator definition is met, between a lower threshold (setting the minimum level of performance required to start earning bonus payments) and an upper threshold (the maximum level of performance when practices stop earning bonus payments). Points are converted into payments according to a formula based on a baseline payment adjusted for relative practice size and condition prevalence. Achievement rates are calculated at the indicator level and take no account of patients’ socioeconomic status. The maximum payment available differs across indicators and broadly reflects the expected amount of effort required to achieve each indicator, rather than the expected health gain.^12^ Practices can exclude (‘exception report’) patients for whom the targets are judged inappropriate (eg, for reasons of frailty or informed dissent), and rates of exception reporting vary widely between practices.^13^

We studied the provision of care incentivised by the QOF during the financial year 2019/2020; in that year, there were 68 indicators covering 26 conditions, and a total of 559 points available.^14^ We restricted our analysis to 35 QOF indicators relating to 11 long-term conditions for which achievement can be measured at the level of individual patients (ie, we excluded practice-level indicators such as disease registers)—see table 1.

**Table 1:** Quality and Outcomes Framework (QOF) indicators included in the study—QOF business rules version 44 (financial year 2019/2020)

Condition	Indicator	Indicator description	Points per indicator	Indicator type*
Atrial fibrillation	AF006	The percentage of patients with atrial fibrillation in whom stroke risk has been assessed using the CHA_2_DS_2_-VASc score risk stratification scoring system in the preceding 12 months (excluding those patients with a previous CHADS_2_ or CHA_2_DS_2_-VASc score of 2 or more)	12	3
	AF007	In those patients with atrial fibrillation with a record of a CHA_2_DS_2_-VASc score of 2 or more, the percentage of patients who are currently treated with anticoagulation drug therapy	12	1
Coronary heart disease	CHD005	The percentage of patients with coronary heart disease with a record in the preceding 12 months that aspirin, an alternative antiplatelet therapy or an anticoagulant is being taken	7	1
	CHD007	The percentage of patients with coronary heart disease who have had influenza immunisation in the preceding 1 August to 31 March	7	1
	CHD008	The percentage of patients aged 79 years or under, with coronary heart disease, in whom the last blood pressure reading (measured in the preceding 12 months) is 140/90 mm Hg or less	12	2
	CHD009	The percentage of patients aged 80 years or over, with coronary heart disease, in whom the last blood pressure reading (measured in the preceding 12 months) is 150/90 mm Hg or less	5	2
Heart failure	HF002	The percentage of patients with a diagnosis of heart failure (diagnosed on or after 1 April 2006) which has been confirmed by an echocardiogram or by specialist assessment 3 months before or 12 months after entering on to the register	6	3
	HF003	In those patients with a current diagnosis of heart failure due to left ventricular systolic dysfunction, the percentage of patients who are currently treated with an ACE-I or ARB	10	1
	HF004	In those patients with a current diagnosis of heart failure due to left ventricular systolic dysfunction who are currently treated with an ACE-I or ARB, the percentage of patients who are additionally currently treated with a beta-blocker licensed for heart failure	9	1
Hypertension	HYP003	The percentage of patients aged 79 years or under, with hypertension, in whom the last blood pressure reading (measured in the preceding 12 months) is 140/90 mm Hg or less	14	2
	HYP007	The percentage of patients aged 80 years or over, with hypertension, in whom the last blood pressure reading (measured in the preceding 12 months) is 150/90 mm Hg or less	5	2
Stroke and transient ischaemic attack	STIA007	The percentage of patients with a stroke shown to be non-haemorrhagic or a history of TIA, who have a record in the preceding 12 months that an antiplatelet agent or an anticoagulant is being taken	4	1
	STIA009	The percentage of patients with stroke or TIA who have had influenza immunisation in the preceding 1 August to 31 March	2	1
	STIA010	The percentage of patients aged 79 years or under, with a history of stroke or TIA, in whom the last blood pressure reading (measured in the preceding 12 months) is 140/90 mm Hg or less	3	2
	STIA011	The percentage of patients aged 80 years or over, with a history of stroke or TIA, in whom the last blood pressure reading (measured in the preceding 12 months) is 150/90 mm Hg or less	2	2
Asthma	AST002	The percentage of patients aged 8 or over with asthma (diagnosed on or after 1 April 2006), on the register, with measures of variability or reversibility recorded between 3 months before or any time after diagnosis	15	3
	AST003	The percentage of patients with asthma, on the register, who have had an asthma review in the preceding 12 months that includes an assessment of asthma control using the three RCP questions	20	3
	AST004	The percentage of patients with asthma aged 14 or over and who have not attained the age of 20, on the register, in whom there is a record of smoking status in the preceding 12 months	6	3
Chronic obstructive pulmonary disease (COPD)	COPD002	The percentage of patients with COPD (diagnosed on or after 1 April 2011) in whom the diagnosis has been confirmed by post-bronchodilator spirometry between 3 months before and 12 months after entering on to the register	5	3
	COPD003	The percentage of patients with COPD who have had a review, undertaken by a healthcare professional, including an assessment of breathlessness using the Medical Research Council dyspnoea scale in the preceding 12 months	9	3
	COPD007	The percentage of patients with COPD who have had influenza immunisation in the preceding 1 August to 31 March	6	1
	COPD008	The percentage of patients with COPD and Medical Research Council (MRC) dyspnoea scale ≥3 at any time in the preceding 12 months, with a subsequent record of an offer of referral to a pulmonary rehabilitation programme (excluding those who have previously attended a pulmonary rehabilitation programme)	2	3
Diabetes mellitus	DM006	The percentage of patients with diabetes, on the register, with a diagnosis of nephropathy (clinical proteinuria) or micro-albuminuria who are currently treated with an ACE-I (or ARBs)	3	1
	DM012	The percentage of patients with diabetes, on the register, with a record of a foot examination and risk classification: (a) low risk (normal sensation, palpable pulses), (b) increased risk (neuropathy or absent pulses), (c) high risk (neuropathy or absent pulses plus deformity or skin changes in previous ulcer) or (d) ulcerated foot within the preceding 12 months	4	3
	DM014	The percentage of patients newly diagnosed with diabetes, on the register, in the preceding 1 April to 31 March who have a record of being referred to a structured education programme within 9 months after entry on to the diabetes register	11	3
	DM018	The percentage of patients with diabetes, on the register, who have had influenza immunisation in the preceding 1 August to 31 March	3	1
	DM019	The percentage of patients with diabetes, on the register, without moderate or severe frailty in whom the last blood pressure reading (measured in the preceding 12 months) is 140/80 mm Hg or less	10	2
	DM020	The percentage of patients with diabetes, on the register, without moderate or severe frailty in whom the last IFCC-HbA1c is 58 mmol/mol or less in the preceding 12 months	17	2
	DM021	The percentage of patients with diabetes, on the register, with moderate or severe frailty in whom the last IFCC-HbA1c is 75 mmol/mol or less in the preceding 12 months	10	2
	DM022	The percentage of patients with diabetes, on the register, aged 40 years or over, with no history of CVD and without moderate or severe frailty, who are currently treated with a statin (excluding patients with type 2 diabetes and a CVD risk score of <10% recorded in the preceding 3 years)	4	1
Dementia	DEM004	The percentage of patients diagnosed with dementia whose care plan has been reviewed in a face-to-face review in the preceding 12 months	39	3
Mental health	MH002	The percentage of patients with schizophrenia, bipolar affective disorder and other psychoses who have a comprehensive care plan documented in the record, in the preceding 12 months, agreed between individuals, their family and/or carers as appropriate	6	3
	MH003	The percentage of patients with schizophrenia, bipolar affective disorder and other psychoses who have a record of blood pressure in the preceding 12 months	4	3
	MH006	The percentage of patients with schizophrenia, bipolar affective disorder and other psychoses who have a record of BMI in the preceding 12 months	4	3
Rheumatoid arthritis	RA002	The percentage of patients with rheumatoid arthritis, on the register, who have had a face-to-face annual review in the preceding 12 months	5	3

* Indicators were classified as: (1) treatment; (2) intermediate outcome; and (3) measurement, assessment or referral.

ACE-I, ACE inhibitor; ARB, angiotensin II receptor blocker; BMI, body mass index; CHADS_2_/CHA_2_DS_2_-VASc, clinical prediction tools for estimating risk of stroke for non-rheumatic atrial fibrillation based on Congestive heart failure, Hypertension, Age, Diabetes mellitus and previous Stroke; CVD, cardiovascular disease; HbA1c, haemoglobin A1c; IFCC-HbA1c, International Federation of Clinical Chemistry and Laboratory Medicine ; IMD, Index of Multiple Deprivation; TIA, transient ischaemic attack.

### Data source

The Clinical Practice Research Datalink (CPRD) Aurum dataset collates electronic health records on patients’ diagnoses, symptoms, referrals, tests and prescriptions generated by participating general practices as part of routine care delivery. Our study used individual-level data on patients with at least one QOF-relevant health condition registered with one of 300 randomly selected (out of approximately 1200) English practices contributing data to CPRD Aurum. Patients included in CPRD Aurum are broadly representative of the English population in terms of age, sex and deprivation.^9 15^

Complete clinical records were extracted from CPRD Aurum for all patients who: were registered with a sampled practice; were aged 18 or over on 31 March 2020 (the end of the financial year 2019/2020); had at least 90 days of prior registration with the practice; and met the eligibility criteria for inclusion in one or more QOF disease registers. A total of 607 126 patients met the criteria and were included in the analysis. Additional data were extracted on patients’ age, sex and an anonymised indicator of the GP practice in which they were registered, as well as the deprivation profile of the neighbourhood in which they resided. The latter was based on the 2019 Index of Multiple Deprivation (IMD), which ranks all 32 844 lower-layer super output areas (LSOAs) in England (administrative areas with a median population of 1500) according to a weighted composite indicator covering seven deprivation domains.^16^ LSOAs were assigned to quintile groups based on their rank, and these quintile groups were mapped to patients’ postcode information by the data provider prior to data release.

The CPRD data were coded according to the QOF business rules for the financial year 2019/2020 (version 44.0). Diagnostic, prescribing and demographic data were used to identify eligible patients for each QOF indicator, measure their individual indicator achievement and determine their exception reporting status. Patients could be eligible for all or a subset of indicators within a given disease area, and they might be exception reported for some eligible indicators but not others. The coded data were compared against national statistics published by NHS England to ascertain coding quality and correct implementation of business rules.

Permission for data usage was obtained from the CPRD Independent Scientific Advisory Committee (protocol number 21_000452).

### Outcomes

The primary outcome was ‘reported achievement’, calculated as the number of patients for whom an indicator was achieved (N) divided by the number of eligible patients who were not exception reported (D). We also analysed two secondary outcomes: ‘population achievement’—the rate of indicator achievement in the full population of eligible patients, including those exception reported (E), calculated as N/(D+E); and the rate of exception reporting in the full population of eligible patients (E/(D+E)).

### Statistical analysis

Logistic regression analysis was used to estimate differences in outcomes across the five IMD quintile groups, from the most deprived (IMD1) to the least deprived (IMD5), separately for each QOF indicator and outcome definition. All SEs were clustered at GP practice level to account for within-practice correlation across observations.

We estimated two different model specifications. Specification 1 included indicators for IMD quintile groups as well as controls for patient age (in 5-year bands), sex and age-sex interactions (the base model). The results of this analysis measure age-sex adjusted inequality in outcomes across deprivation groups in England (‘overall inequality’). This adjustment ensures that differences in age structure across practices or deprivation groups do not drive the reported inequalities. Inequalities may arise from differences in treatment patterns within practices or differences in clinical performance across practices that correlate with characteristics of the registered population under care. To delineate these effects, Specification 2 controlled additionally for GP practice fixed effects. Although fixed effects do not directly measure practice attributes, they act as a statistical control, capturing differences in both observed and unobserved time-invariant practice characteristics (eg, staffing, clinical protocols). The resulting estimates reflect inequality among patients treated in the same practice (‘within practice inequality’) and thus constitute more direct evidence of inequality that is subject to GP practices’ clinical and managerial choices.

Estimated coefficients on the logit scale were translated into average marginal effects with associated 95% CIs to aid comparison in terms of rates of outcomes (expressed in percentages or positive outcome per 100 patients). Inequality was measured as the difference between rates in IMD1 (most deprived) and IMD5 (least deprived). Negative values denote higher achievement rates for patients living in the most deprived areas, and vice versa. This ‘inequality gap’ metric was selected over alternative measures of inequality for ease of interpretation.

## Results

A total of 607 126 patient records were included in the analysis. On average, each patient was eligible for inclusion in 3.2 (of 35) QOF indicators (median=2; IQR=1–5), with those living in the most deprived 20% of neighbourhoods (IMD1) on average being eligible for more QOF indicators than those in the least deprived 20% of neighbourhoods (IMD5) (mean=3.5 vs 2.9). Descriptive statistics for the patient population overall and by indicator are reported in online supplemental tables 1 and 2 and unadjusted rates of reported achievement, population achievement and exception reporting by deprivation quintile are reported in online supplemental tables 3–5.

### Overall results

Figure 1 reports age-sex adjusted achievement and exception reporting rates by deprivation quintile group pooled over all patient-indicator combinations. There was no clear socioeconomic gradient in overall reported achievement, with an absolute gap of 0.1 percentage points between the most (IMD1) and least deprived (IMD5) quintile that was not statistically significant (95% CI −0.5% to 0.3%). In contrast, population achievement rates decreased monotonically with deprivation, with rates 1.4 percentage points lower in the most deprived (IMD1) compared with the least deprived (IMD5) quintile (95% CI −1.9% to −1.0%). The different patterns for reported and population achievement rates are partly explained by differences in exception reporting, for which rates generally increased with deprivation (11.7% in most deprived vs 9.9% in the least deprived quintile; inequality gap: −1.8%, 95% CI −2.1% to −1.5%).

**Figure 1:**
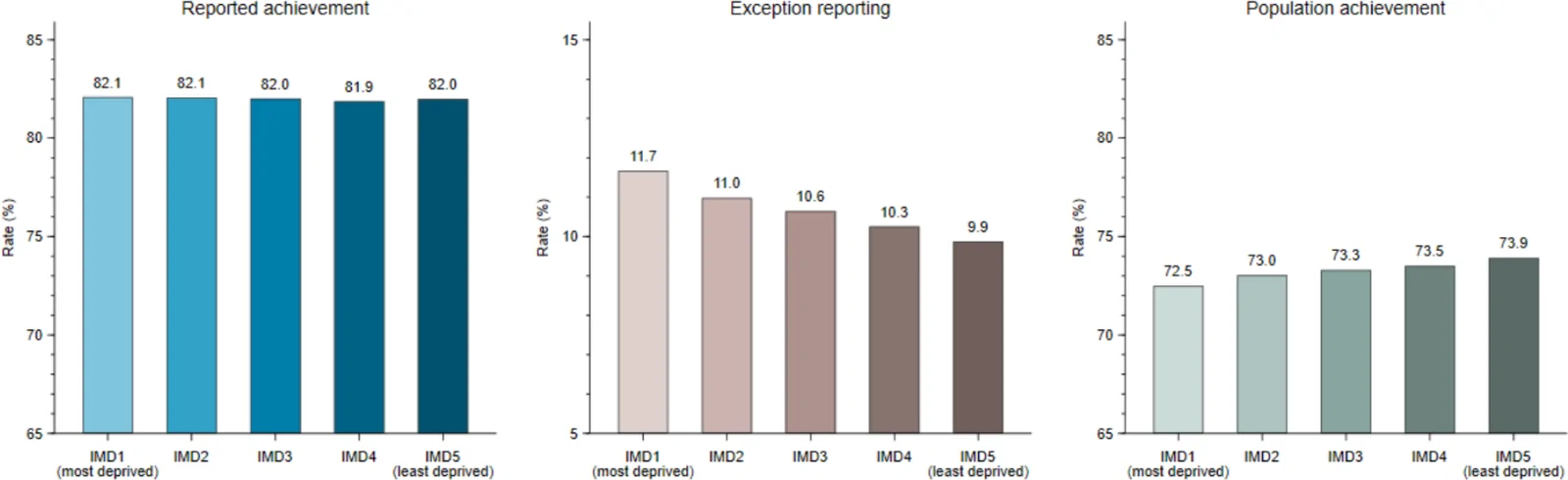
Rates of pooled reported achievement, exception reporting and population achievement, adjusted for patient age and sex. IMD, Index of Multiple Deprivation.

### Results by indicator

Figure 2 shows the absolute gap in rates of reported achievement, exception reporting and population achievement based on Specification 1 (adjusted for age and sex) by indicator. Inequality gaps in adjusted reported achievement rates ranged from −3.8% (95% CI −7.1% to −0.6%) (CHD5: anticoagulant therapy for patients with CHD) to 3.8% (95% CI −0.2% to 7.7%) (AST002: measures of variability or reversibility recorded in patients with asthma). There was no clear socioeconomic pattern across indicators, with five having significantly higher achievement rates in the least deprived group, two having significantly higher achievement rates in the most deprived group, and 28 having differences between the most and least deprived groups that were not statistically significantly different from zero. There were also no discernible socioeconomic patterns across disease areas.

**Figure 2:**
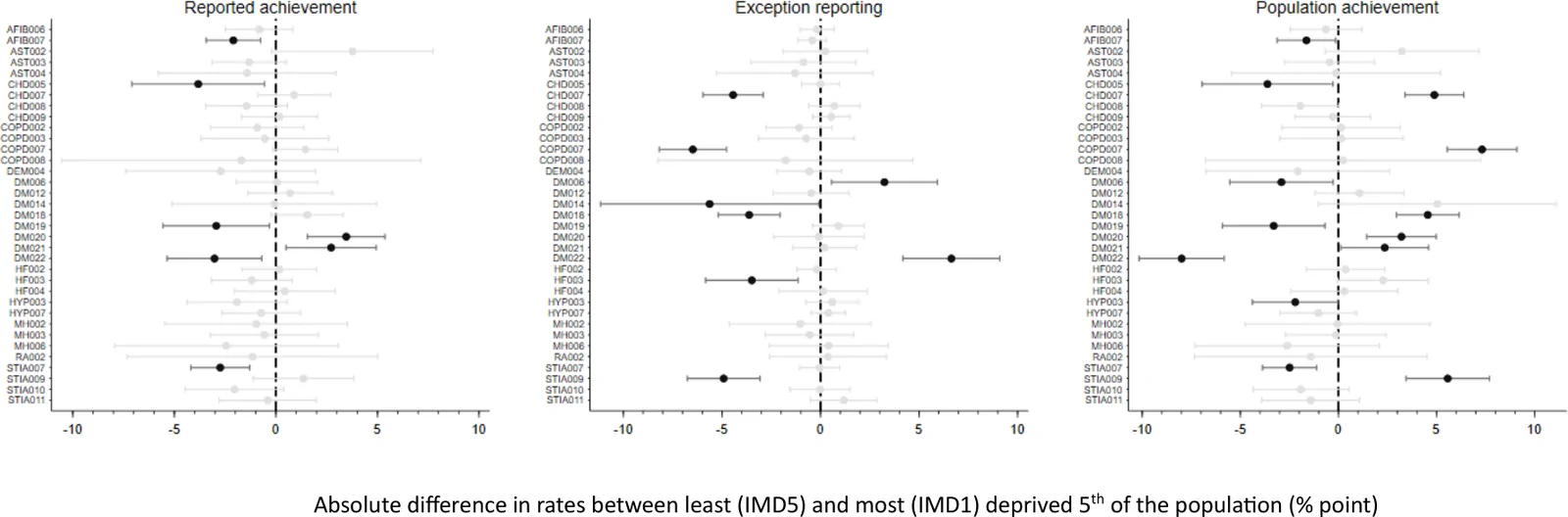
Inequality gap in achievement (IMD5 minus IMD1) by indicator, adjusted for patient age and sex. Differences that are not statistically significant (at alpha=5%) are greyed out. IMD, Index of Multiple Deprivation.

Gaps for exception reporting rates ranged from −6.5% (95% CI −8.2% to −4.8%) for COPD007 (influenza vaccination for patients with COPD) to 6.6% (95% CI 4.2% to 9.1%) for DM022 (statin prescription for patients with diabetes without cardiovascular disease and low levels of frailty); gaps for population achievement rates mirrored exception reporting rates and ranged from −8.0% (95% CI −10.2% to −5.8%) for DM022 to 7.3% (95% CI 5.5% to 9.1%) for COPD007. This inverse relationship is to be expected since patients who achieved the indicator cannot, as per QOF business rules, be exception reported, nor would it be clinically plausible to do so. Consequently, higher rates of exception reporting in the most deprived group contribute to higher population achievement rates in the least deprived group. As with reported achievement, there was no systematic socioeconomic pattern in exception reporting or population achievement rates across indicators. However, there were significantly higher prescribing rates for people living in more deprived areas for anticoagulants (AF007, CHD005 and STIA007), ACE-inhibitors (DM006) and statins (DM022). In contrast, all four indicators relating to influenza vaccination (CHD007, COPD007, DM018 and STIA009) and two indicators relating to glycaemic control (DM020, DM021) had higher achievement rates for patients living in less deprived areas.

The inclusion of GP practice fixed effects (Specification 2) revealed more consistent socioeconomic patterns (figure 3). Eight of the 35 indicators demonstrated statistically significant gaps for reported achievement rates; in all cases achievement rates were significantly higher for patients living in less deprived areas, with the biggest gap (4.3%; 95% CI 3.1% to 5.4%) for DM020 (HbA1c≤58 mmol/mol for patients with diabetes). Similarly, for population achievement 13 indicators had significantly higher achievement rates for patients living in less deprived areas, with the biggest gap (6.0%; 95% CI 4.4% to 7.6%) for COPD007. One indicator, DM022 (statin treatment for patients with diabetes without a history of cardiovascular disease) had a significantly lower population achievement rate for patients in the least deprived compared with the most deprived quintile (−3.2%; 95% CI −4.6% to −1.8%). These patterns frequently mirrored those for exception reporting, for which rates were significantly higher in the most deprived compared with the least deprived areas for 13 indicators and significantly lower for one indicator. All six indicators with higher population achievement rates in the least deprived group under Specification 1 followed a similar pattern under Specification 2, as did four indicators relating to recording and reviewing activities (AST002, AST003, COPD003 and DM012) and three indicators relating to blood pressure control (CHD008, HYP003—which had higher achievement rates in the most deprived group under Specification 1—and STIA10).

**Figure 3:**
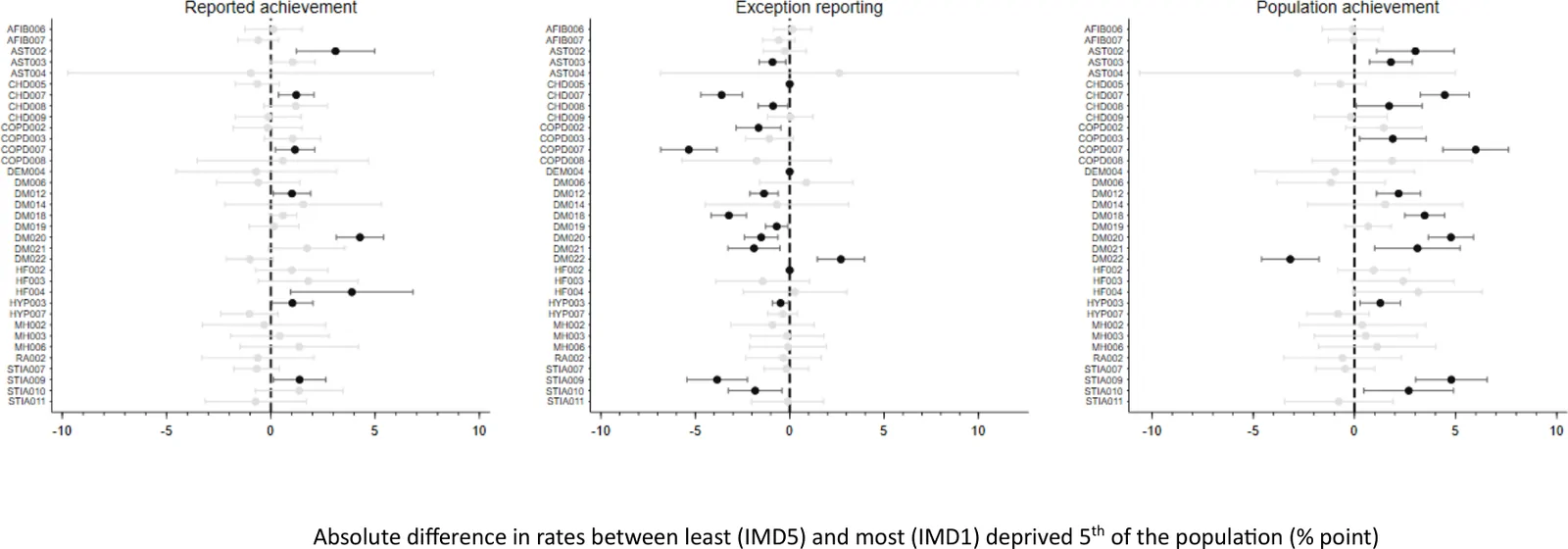
Inequality gap in achievement (IMD5 minus IMD1) by indicator, adjusted for patient age and sex and general practitioner practice fixed effects. Differences that are not statistically significant (at alpha=5%) are greyed out. IMD, Index of Multiple Deprivation.

Figure 4 plots within-practice inequality in reported achievement against overall inequality metrics for all 35 indicators. The 45° line denotes the line of equality between the two definitions of inequality. Most indicators lie above the 45° line, implying that within practice inequality typically exceeds overall inequality across a range of incentivised care processes.

**Figure 4:**
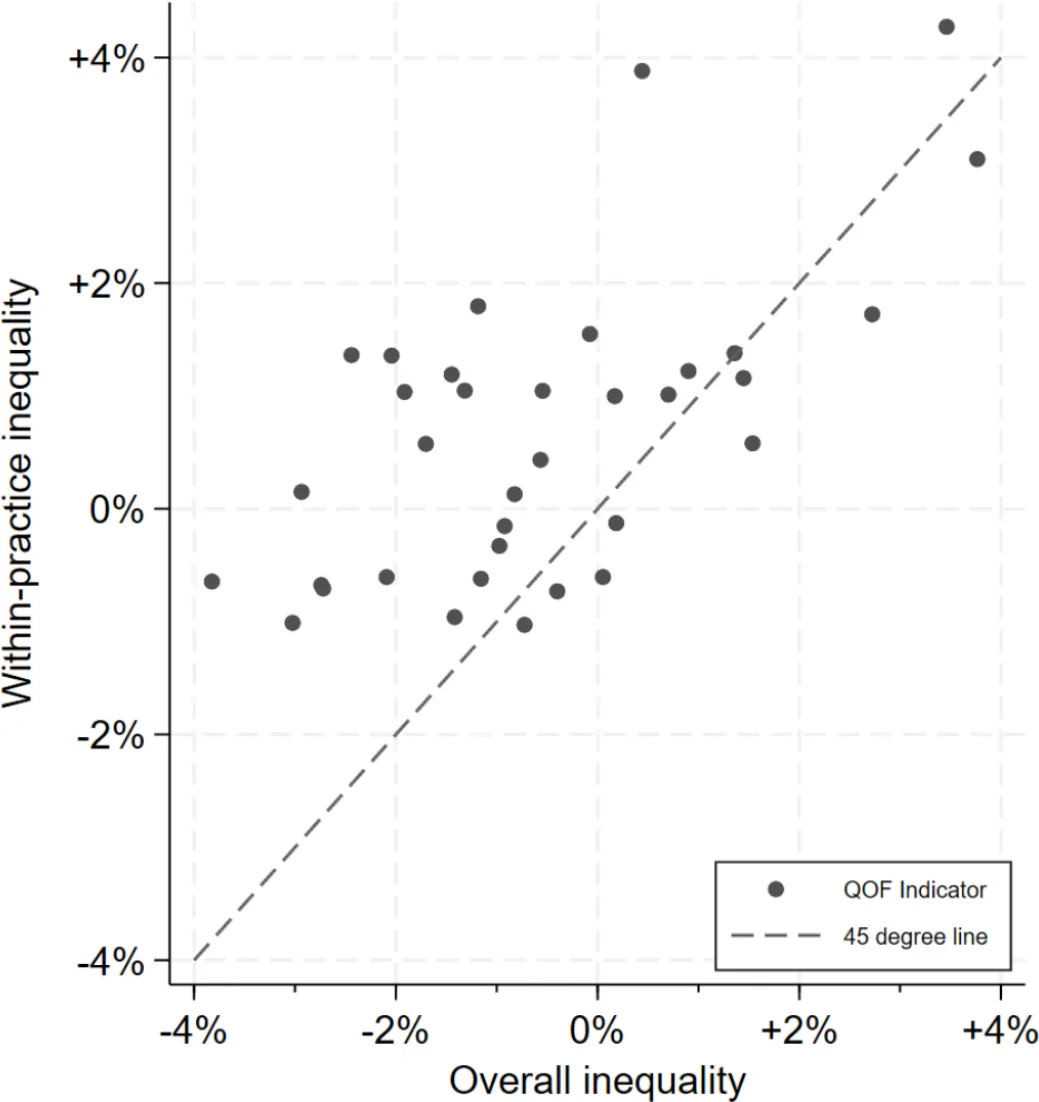
Overall inequality and within-practice inequality by indicator (reported achievement). QOF, Quality and Outcomes Framework.

## Discussion

### Main findings

We used routinely collected electronic primary care records to estimate socioeconomic variations in achievement rates for 35 quality of care indicators, incentivised under the QOF. When overall results were pooled across all indicators, there was no clear socioeconomic gradient in *reported* achievement for individual patients, that is, achievement after practices have removed patients judged to be inappropriate for a given indicator. However, there were socioeconomic gradients for exception reporting and for *population* achievement (ie, achievement for all patients with a given condition, whether the practice deemed the indicator appropriate or not), with patients in more deprived areas generally being more likely to be excluded and less likely to meet the quality targets.

Across individual indicators at the cohort level, there was no consistent relationship between area deprivation and achievement rates (either reported or population) or exception reporting rates, with some indicators having significantly higher achievement rates for patients in the most affluent areas and others having higher rates for patients in the most deprived areas. However, particular types of activity did tend to have higher achievement rates and lower exception reporting rates for patients in more deprived areas (eg, prescribing of anticoagulants), whereas these patterns were reversed for other types of activity (influenza vaccination, glycaemic control in diabetes).

More consistent socioeconomic patterns emerged after controlling for GP practice fixed effects. For patients living in more deprived areas, reported achievement tended to be lower for eight (of 35) indicators, population achievement was lower for 13 indicators, and exception reporting was higher for 13 indicators. Only two results favoured more deprived patients, with higher population achievement and lower exception reporting rates for statin prescribing in patients with diabetes and without cardiovascular disease. Again, particular types of activity had lower achievement rates and higher exception reporting rates for patients in more deprived areas: care reviews, influenza vaccination and intermediate outcomes (glycaemic control and blood pressure control). However, this pattern was not apparent for indicators relating specifically to patients aged over 80 (CHD009, HYP007, STIA011), for whom blood pressure targets are higher.

The difference in results with and without practice fixed effects suggests that, at the national level, there appears to be no consistent disadvantage for people living in more deprived areas in terms of receiving a QOF incentivised intervention, but *within practices* people living in more deprived areas are less likely to receive the intervention for a minority of QOF indicators. This apparent contradiction could be explained by practices with higher proportions of patients from deprived areas reporting better performance on some QOF indicators on average than practices with lower proportions; this may offset within-practice inequalities to minimise inequalities at the national level. Previous practice-level analyses suggest that practices located in more deprived areas tend to score *fewer* points on average across the whole QOF,^9^ but points allocations are based on reported achievement rates for the whole practice population relative to payment thresholds and are unadjusted for age and sex, and therefore follow different patterns to adjusted reported and population achievement rates. These differences arise in part because the most deprived areas in England have proportionately more people in younger age groups and less people in higher age groups than the least deprived areas^17^ and achievement rates vary with age. Future analyses comparing practices with high and low concentrations of deprived patients, adjusted for age, could help identify practice characteristics or organisational approaches associated with more equitable delivery of care.

Within-practice variation can arise when social inequality creates greater barriers for more deprived patients in seeking, receiving and benefiting from healthcare.^18^ Even under a universal system, people from poorer backgrounds are more likely to delay seeking care and not attend appointments out of preference or due to competing priorities, to experience communication and navigation barriers when engaging with services, and to have complex social and medical needs and reduced capacity for self-management.^3^ These factors are likely to have contributed to both the higher rates of exclusion^19^ and lower rates of population achievement for more deprived patients, particularly for intermediate outcomes indicators such as glycaemic and blood pressure control that require active patient participation. In contrast, indicators primarily driven by prescribing decisions had weaker or reversed gradients and higher overall rates of achievement. This suggests that inequalities are more likely to arise where achievement depends on repeated contact, sustained adherence or behaviour change, rather than on a single clinical action. Notably, indicators applying to patients aged over 80 did not show consistent socioeconomic gradients. This may partly reflect less demanding clinical targets under the QOF for older patients, as well as differences in consultation frequency, availability of time or prioritisation of care in later life. These findings highlight the importance of interpreting inequality patterns in relation to indicator specifications and target populations.

It should be noted that our results relate to processes of care and intermediate outcomes and are likely to underestimate inequalities in final outcomes, such as emergency hospital admission and mortality. There is evidence that better QOF performance is associated with better patient outcomes for some activities but not others,^8^ and that the relationship between quality of primary care and outcomes is influenced by patient characteristics and local secondary care provision.^20^ Even if practices deliver equal levels of care to all their patients, the resulting health benefits are unlikely to be distributed equally due to differences in treatment adherence and response. In addition, although social differences in quality of care as measured by achievement of QOF indicators appear to be small, the probability of developing the relevant conditions is substantially higher for people living in more deprived areas.^17^ At the population level, inequalities in outcomes will therefore be substantial, mainly due to the social gradient in prevalence of long-term conditions, over which practices have limited control.

### Strengths and limitations

To minimise misclassification, we used a large, representative database of electronic patient records drawn from GP practices in England linked to a standard measure of area deprivation through individual patient postcodes, rather than through practice location. For outcomes, we used quality indicators from an established national pay-for-performance programme, limiting systematic biases in how practices diagnose conditions and deliver relevant care. However, the study is descriptive and therefore cannot identify causal mechanisms, and has several limitations due to its dependence on administrative data collected under a financial incentive scheme. In particular, we were reliant on the accurate and complete recording of clinical activity, and any systematic differences in how practices record achievement for people from different socioeconomic backgrounds will bias our results. We also focused on single conditions included in the QOF and have not examined interactions between them or considered potential impacts on non-incentivised aspects of care. Quality of care for patients with multimorbidity may follow different socioeconomic patterns to those we describe here. Given data limitations, we were unable to explain individual-level variation in achievement within practices (including the roles of patient preferences, clinical judgement, multimorbidity or nuanced social circumstances) or the reasons for higher exception reporting (eg, non-response to invitations, informed dissent or strategic misreporting by practices under target-based schemes^21^). Finally, the data do not allow direct examination of practice-level characteristics such as staffing levels, appointment availability or access to community and secondary care services, which may influence both achievement and exception reporting rates.

## Conclusions

Our results suggest that patients living in more deprived areas are less likely to receive some interventions for common long-term conditions than patients from less deprived areas attending the same practice. These differences are generally small (less than 5%) but significant, and may be missed by practice-level analysis. Policy and system responses to address these inequalities could include: addressing access barriers—for example, through alternative consultation arrangements and tailored communication approaches^22^; revision of the Carr-Hill formula to better reflect socioeconomic deprivation and multimorbidity in allocating capitation payments^23^; and adjustment of incentive structures to reflect higher social barriers in more deprived areas—for example, through equity-weighted incentives or rebalancing payments to favour activities with greater benefits for more deprived patients. The impact of such changes would need to be evaluated through systematic monitoring of within-practice inequalities in quality of care and of the socioeconomic distribution of health outcomes related to primary care.

## Supplementary material

10.1136/jech-2025-225416 online supplemental file 1

## Data Availability

Data may be obtained from a third party and are not publicly available.
